# Chiral Materials for Optics and Electronics: Ready to Rise?

**DOI:** 10.3390/mi15040528

**Published:** 2024-04-15

**Authors:** Seo-Hyeon Ham, Moon Jong Han, Minkyu Kim

**Affiliations:** 1 Department of Chemical Engineering, Dankook University, Yongin 16890, Republic of Korea; ham2992@naver.com; 2 Department of Electronic Engineering, Gachon University, Seongnam 13120, Republic of Korea

**Keywords:** chirality, optics, electronics

## Abstract

Chiral materials have gained burgeoning interest in optics and electronics, beyond their classical application field of drug synthesis. In this review, we summarize the diverse chiral materials developed to date and how they have been effectively applied to optics and electronics to get an understanding and vision for the further development of chiral materials for advanced optics and electronics.

## 1. Introduction

**Figure 1:**
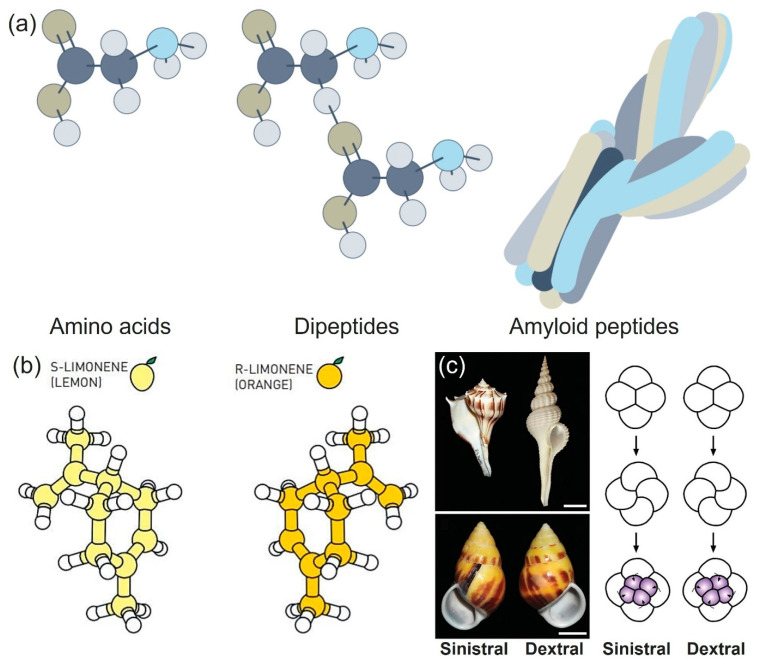
Chirality in nature. (**a**) Chemical structure of amino acids, dipeptides, and amyloid peptides. dark blue: carbon, grey: hydrogen, light brown: oxygen, light blue: nitrogen. Reproduced with permission from [1]. Copyright © 2022 Nature Photonics. (**b**) Chemical structure of (*S*)- and (*R*)-limonene, respectively, Reproduced with permission from [3]. Copyright © Johan Jarnestad/The Royal Swedish Academy of Sciences. (**c**) Sinistral and dextral structure of snail. Reproduced with permission from [4], Copyright © 2008 Nature.

Chiral materials, including amino acids and proteins, play a significant role in biological processes. Chiral molecules are involved in important biological processes, but their mirror images exhibit different biological properties (Figure 1a) [1]. So, what is ‘chirality’? Chirality refers to the characteristic of a molecule or object having an inherent asymmetry, preventing it from aligning perfectly with its mirror image through rotation and translation. Enantiomers are pairs of molecules with identical chemical structures but differing spatial arrangements of atoms, creating mirror-image relationships that cannot be superimposed. Enantiomers possess very similar energy levels, but they can show different properties [2]. For instance, a (*S*)-limonene molecule smells like lemon, while (*R*)-limonene smells like orange (Figure 1b) [3]. Chirality in nature can be easily found in structures such as snail shells (Figure 1c) [4]. To date, diverse chiral materials have been reported, from simple organic chiral molecules to complex chiral nanoparticles (NPs) [5,6], polymers [7], and micron-sized chiral structures [1,8,9,10,11]. Accordingly, the types of chirality of chiral materials have continuously increased in number, as will be described below.

The presence of chirality in an object results in unique properties that achiral objects cannot show, such as circularly polarized light reflection, emission, or transmission, chiral-induced spin selectivity, etc. [12]. Due to these unique properties, chiral materials have drawn burgeoning interest from the optics and electronics fields, beyond their classical application field of drug synthesis. While chiral materials have been increasingly applied to optics and electronics for unique purposes and/or higher performance, limited kinds of chiral materials have been utilized for these fields thus far, indicating that there is plenty of room for chiral materials to further advance optics and electronics. In this review, we will take a look at the various chiral materials developed to date and how they have been effectively applied to optics and electronics to get insight into the further development of chiral materials for advanced optics and electronics. The primary focus of our review is to present the diverse research and the paramount potential of chiral materials, specifically their optical and electronic applications.

## 2. Classification of Chiral Materials Depending on the Chemical Composition

### 2.1. Organic Chiral Materials

**Figure 2:**
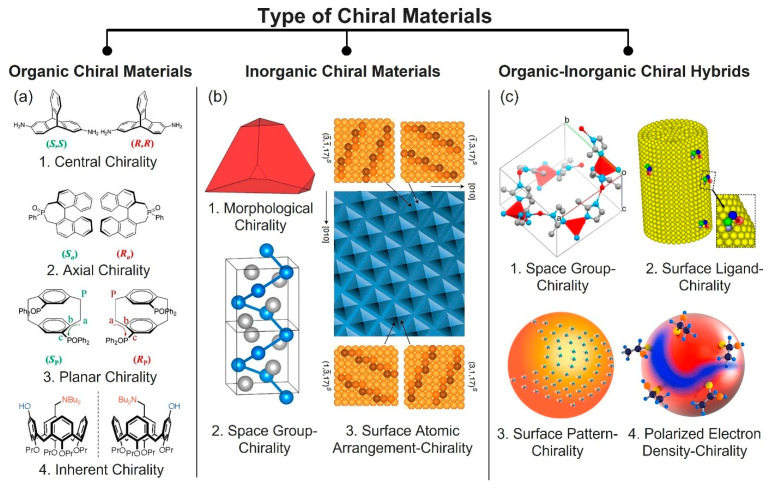
Classification of chiral materials. (**a**) Organic chiral materials: (1) central chirality, (2) axial chirality, (3) planar chirality. Reproduced with permission from [13]. (4) inherent chirality. Reproduced with permission from [15]. Copyright © 2007 American Chemical Society. (**b**) Inorganic chiral materials: (1) morphological chirality. Reproduced with permission from [27]. Copyright © 2017 American Chemical Society. (2) space-group chirality. Reproduced with permission from [28]. Copyright © 2020 Springer Nature. (3) surface atomic arrangement-chirality. Reproduced with permission from [29]. Copyright © 2020 Springer Nature. (**c**) Organic-inorganic chiral materials: (1) space-group chirality, Reproduced with permission from [30]. Copyright © 2010 Springer Nature. (2) surface-ligand chirality, (3) surface pattern chirality, (4) polarized electron density chirality. Reproduced with permission from [27]. Copyright © 2017 American Chemical Society.

**Figure 3:**
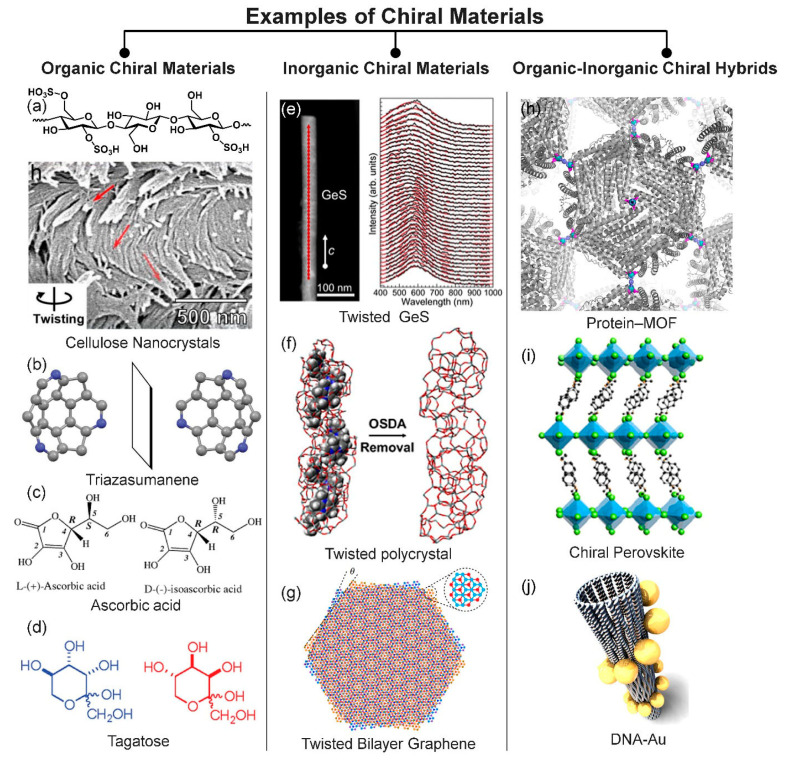
Examples of chiral materials. (**a**–**d**) Organic chiral materials: (**a**) cellulose nanocrystals. Reproduced with permission from [11]. Copyright © 2021 American Chemical Society. (**b**) triazasumanene. Reproduced with permission from [16]. Copyright 2012 Springer Nature. (**c**) ascorbic acid. Reproduced with permission from [22]. Copyright © 2009 American Chemical Society, and (**d**) tagatose [20]. Reproduced with permission from [19]. Copyright © 2011 American Chemical Society. (**e**–**g**) Inorganic chiral materials: (**e**) twisted GeS. Reproduced with permission [31]. Copyright © 2019 Springer Nature. (**f**) twisted polycrystal. Reproduced with permission [32]. Copyright © 1999-2024 John Wiley & Sons, and (**g**) twisted bilayer graphene. Reproduced with permission from [33]. Copyright © 2020 Springer Nature. (**h**–**j**) Organic-inorganic chiral materials: (**h**) protein-MOF. Reproduced with permission [34]. Copyright © 2013 American Chemical Society, (**i**) chiral perovskite. Reproduced with permission from [35]. Copyright © 2019 American Chemical Society, and (**j**) DNA-Au. Reproduced with permission from [35]. Copyright © 2017 American Chemical Society.

For organic chiral materials, chiral molecules can be categorized into four types (Figure 2a): [13,14]. The first type is an organic molecule possessing central chirality. This type of chiral organic molecule possesses one or more stereocenters. The second and third types are the chiral organic molecules having a stereogenic axis (axial chirality) and a stereogenic plane (planar chirality), respectively. Last, the inherent bent of a molecule can also result in chirality (inherent chirality) [15,16]. Organic chiral materials possess a strong electron-phonon coupling, an interaction between an electron and lattice vibration [17]. The electrons in chiral nanomaterials are placed in an asymmetric chiral potential field, and their trajectories lead to orbital angular momentum. The orbital effect offers a more abundant opto-magneto-electric coupling effect [12]. Hence, it can influence the magnitude of spin polarization. Furthermore, the chirality-generated orbital angular momentum can influence spin polarization. Overall, for organic chiral materials, the couplings among the electron spin, phonon, and orbit can be influenced by each other, and accordingly can be amplified. This allows the existence of a chirality-induced spin selection effect and circularly polarized light reflection, emission, or transmission. The typical chiral organic materials include sugars [18,19,20], vitamins [21,22], amino acids, peptides, proteins, DNA (deoxyribonucleic acid), and cellulose nanocrystals (CNC) (Figure 3a) [1]. Chiral organic materials have been effectively applied to a myriad of fields, including optical data storage, liquid crystal (LC) displays, and energy storage [23,24,25,26].

### 2.2. Inorganic Chiral Materials

For inorganic chiral materials, chirality can come from a crystal’s shape, the chiral space group of a crystalline structure, or a chiral atomic arrangement on the surface (Figure 2b) [27,28,29,30]. Any asymmetrically-shaped inorganic particles can be classified into chiral organic materials based on this definition [31,32,36]. Regarding crystalline structure, there are sixty-five chiral space groups, known as the Sohncke groups, and inorganic materials possessing any of the sixty-five chiral space groups are chiral inorganic materials [36]. One typical example of an inorganic chiral material that has a chiral crystalline structure is quartz crystal [37]. One example of surface chirality is research that showed that a chiral atomic arrangement on the surface of Cu(100) RABiTS can also create chirality [29]. Very interestingly, while individual particles are achiral, the chirality can be also generated by a twisted assembly of achiral particles, such as is found in twisted bi-layer graphene (Figure 3b) [37] or carbon nanotubes [38], which have been effectively utilized in vast application fields including electronics, optics, and biomedicine [39,40].

### 2.3. Organic-Inorganic Hybrid-Chiral Materials

The chiralities of organic-inorganic hybrids can be divided into four types (Figure 2c): (i) chirality stemming from the chiral space group of the crystal [30], (ii) chirality that originates in the attachment of chiral ligands, (iii) chirality created from a chiral pattern of chiral/achiral ligands, and (iv) chirality generated by the asymmetrically polarized electron density of the core, which is induced by the adsorption of chiral ligands or the chiral patterns of chiral/achiral ligands on the surface of NPs [27]. Thus, in order to fundamentally understand the origins of an organic-inorganic hybrid’s chirality, it is vital to thoroughly investigate its intermolecular and interparticle interactions, surface chemistries, and nanoscale shapes [27]. A chiral metal-organic framework (MOF) is one typical type of chiral organic-inorganic hybrid (Figure 3c). MOFs are composed of inorganic joints and organic linkers and are self-assembled into two- or three-dimensional porous crystalline networks. MOFs have numerous advantages, such as high porosity, controllable surface area, tunable chemical and physical properties, and the feasibility of large-scale production [41]. For these reasons, MOFs have drawn considerable interest. Among diverse MOFs, chiral MOFs have received great attention as promising nano-porous materials for enantioselective membranes, sensing, or catalysts [30]. One of the grand challenges in synthesizing chiral MOFs is forming the chiral structures from achiral precursors and homochirality [41,42]. Most widely, chiral MOFs have been synthesized by directly employing chiral molecules as building blocks [34,39]. This method is straightforward, but there is a limited pool of chiral precursors. In sharp contrast, creating chiral MOFs from achiral precursors has strong benefits that could prevail over the conventional method, in term of the abundance of achiral precursors and cost-effectiveness [41,43]. However, this method has suffered difficulties in the process of synthesizing chiral MOFs, such as controlling chirality [44]. Recently, Kim et al. developed methods for synthesizing a chiral MOF, namely a zeolitic imidazolate framework, from achiral precursors by utilizing CNC, one of the chiral biopolymers [30]. The synthesized chiral MOF demonstrates its ability to chemo-resistively detect the enantiomers. This is the very first example showing the ability to use chiral MOFs as enantioselective electronic sensors, to the best of our knowledge. The unique feature of organic-inorganic hybrid chiral materials compared to chiral organic or inorganic materials is that they can exhibit the synergetic properties of organic and inorganic parts [45,46,47]. For instance, the DNA-Au hybrid can show chiral plasmonic through highly delocalized plasmonic states (Figure 3c) [35]. Also, as another example of chiral organic-inorganic hybrids, chiral perovskites have gained rapidly-growing interest due to their combined unique features, including chiroptical properties such as their absorption or emission of circular polarized light (Figure 3c) [48,49].

## 3. Optical Applications of Chiral Materials

**Figure 4:**
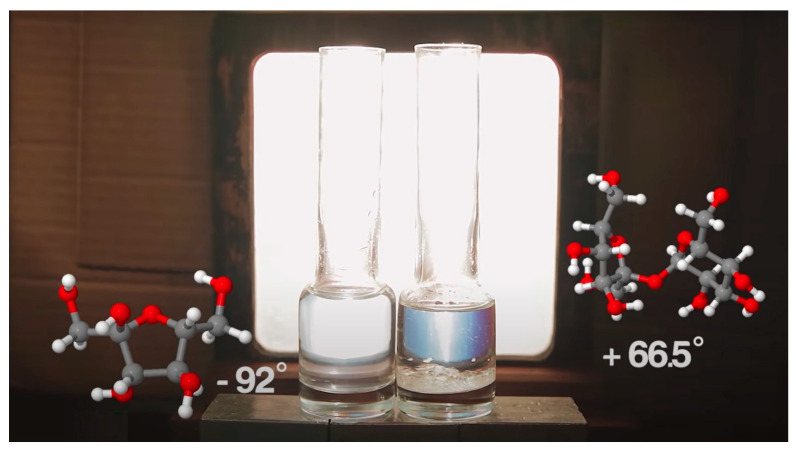
Intrinsic optical activity of chiral materials. The photograph of a fructose solution (left) and sucrose (right) in between linear polarizing filters, presenting different visible colors by optical rotation of chiral materials with different specific optical rotations. Reproduced with permission from [50]. Copyright © Royal Society of Chemistry 2012.

One of the intrinsic properties of chiral molecules is that they can show optical activity, more specifically optical rotation. For instance, when linearly-polarized light reaches chiral molecules, the chiral molecules rotate in the direction of the linearly-polarized light, with different degrees of rotation depending on the kinds of chiral molecules (Figure 4) [50]. Based on the unique fundamental optical properties of chiral materials, up to date, diverse optical applications of chiral materials have been developed, including (1) light controls and (2) sensing.

### 3.1. Light Controls

**Figure 5:**
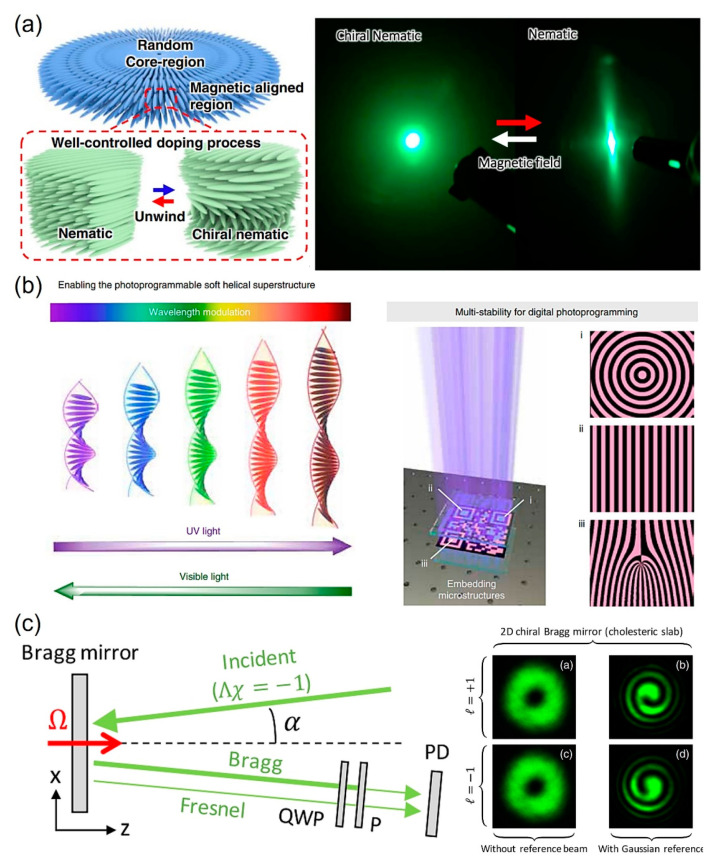
Chiral materials employed for light controls. (**a**) Schematic for the formation of the nematic structure of bacterial CNC/magnetic NP composite by magnetic field-assisted drying that replaces chiral nematic structure formation of bacterial CNC dried without magnetic field (**left**). The light scattering of bacterial CNC film and bacterial CNC/magnetic NPs films (**right**) The red and white arrow indicate the film dried on and without the magnetic field, respectively. Reproduced with permission from [51]. Copyright © 2022 Springer Nature. (**b**) Pitch distance regulation of photo-switchable chiral diarylethene-doped LC via UV/Vis light emission and patterned photo-switchable chiral diarylethene-doped LC formed by light passing photo mask on the doped LC. Reproduced with permission from [52]. Copyright © 2022 Springer Nature. (**c**) Schematic for generation of the dynamic geometric phase of light. QWP: quarter-wave plate; P: polarizer. The Bragg mirror is rotated at an angular frequency Ω = 20°/s. Reproduced with permission from [55]. Copyright © 2016 American Physical Society.

Tsukruk and co-workers reported that controlling light pathways can be made possible using a magnetic NP decorated bacterial CNC (Figure 5a) [51]. In this research, bacterial CNC was prepared from bacterial cellulose, then mixed with Fe_3_O_4_ NPs, resulting in a magnetic bacterial CNC composite. The composite self-assembled into a chiral nematic structure without any magnetic field. composite self-assembled into a nematic structure when the it was dried on the magnetic field. Isotropic light scattering was observed when light passed through the chiral nematic structure, while anisotropic light scattering was shown when light passed through the nematic structure. Meanwhile, Feringa and co-workers demonstrated that the wavelength of reflected light can be tuned by employing a photo-switchable chiral material (Figure 5b) [52]. They first synthesized a photo-switchable chiral diarylethene compound that can change its geometrical figure from a linear to a bent structure. Then, a photo-responsive helical LC was created by adding the photo-switchable chiral diarylethene compound into a chiral nematic LC. As the prepared chiral diarylethene compound-doped LC was continuously exposed to UV light, the linear structure of the diarylethene compound was continuously transformed into a bent structure with an increasing bending angle, leading to an increase in the pitch distance of the LC compound and vice versa, and thus, to a color change. Of the chiral photo-switchable materials, azobenzene is the most often utilized chiral photo-switch on chiral materials and their applications optics. Derivatives of azobenzene are readily available, chemically stabile, and resistant to fatigue. To date, numerous research studies have been performed on creating high-performance photo-switches utilizing azobenzene. Research on materials that can react in more normal conditions and attain quantum yield was conducted by Herges and co-workers [53]. The diazocines they produced show remarkably high conversion rates, enhanced quantum yield, and visible range wavelength switching. Additionally, diazocines demonstrate great thermodynamic stability, which is advantageous for applications such as photo-pharmacology and mechano-sensing. LC superstructures with chiral photo-switches were also reported. Tamaoki and co-workers synthesized azobenzene dopants, which are asymmetric dimeric chiral compounds, to create photon-mode chiral switches for the reversible tuning of self-assembled helical superstructures [54]. Photoisomerization of the azobenzene units under UV light irradiation causes exceptional switching of the helical twisting power and cholesteric pitch. This phenomenon happens as a result of disrupting the helical orientation of molecules in cholesteric LCs formed by elongated cybotactic smectic clusters. Asymmetric dimeric chiral compounds offer significant potential for use as light-driven molecular motors since their photochemical switching enables more control over the rotating motion of micro-sized objects on the surface of CLCs at low doping levels. Last, Brasselet and co-workers presented findings that the dynamic geometric phase of light can be achieved using chiral nematic LCs (Figure 5c) [55]. Specifically, the planar chiral nematic LC film was first prepared. Next, the film was rotated. By emitting light into this chiral Bragg mirror, the dynamic geometric phase of light could be achieved. Specifically, the reflected vortex beam maintained its azimuthal dependence for the amplitude as shown in Figure 5c, in which spiral patterns of opposite handedness were achieved, as illustrated in Figure 5c, with a Gaussian reference. Additionally, Lu and co-workers presented large bending deformations of twisted nematic LC molecules as actuators in response to heat. These molecules exhibit both elastic and optical anisotropies [56]. Furthermore, Yu and co-workers summarized their work on a structural color-based physically-unclonable functions including chiral materials [57]. For instance, a study by Geng and co-workers showed the great promise of utilizing LC phase materials with comparable structural colors as optical PUF anti-counterfeiting labels [58]. Leveraging the dynamic reaction of LC to different light stimuli enables the structure of monodisperse cholesteric LC microspheres to produce a range of structurally changeable color patterns. They highlighted that the optical quality of the patterns is improved, which enhances their usefulness in anti-counterfeiting applications.

### 3.2. Sensing

Two drugs/agrochemicals having identical chemical formulas but that are enantiomerically different can exhibit efficacies or invalid/side effects [1,59]. Therefore, accurate identification of enantiomers is pivotal in manufacturing drugs as well as agriculture. For the high throughput detection of chiral molecules, various methods have been developed, including column chromatography, nuclear magnetic resonance (NMR) spectroscopy, circular dichroism, and fluorescent sensors accompanied with visible color changes [41,60]. Among these approaches, circular dichroism and fluorescent sensors have strong advantages over the other methods, including the potential of quick colorimetric optical confirmation, short measurement time, and ease of sample preparation.

**Figure 6:**
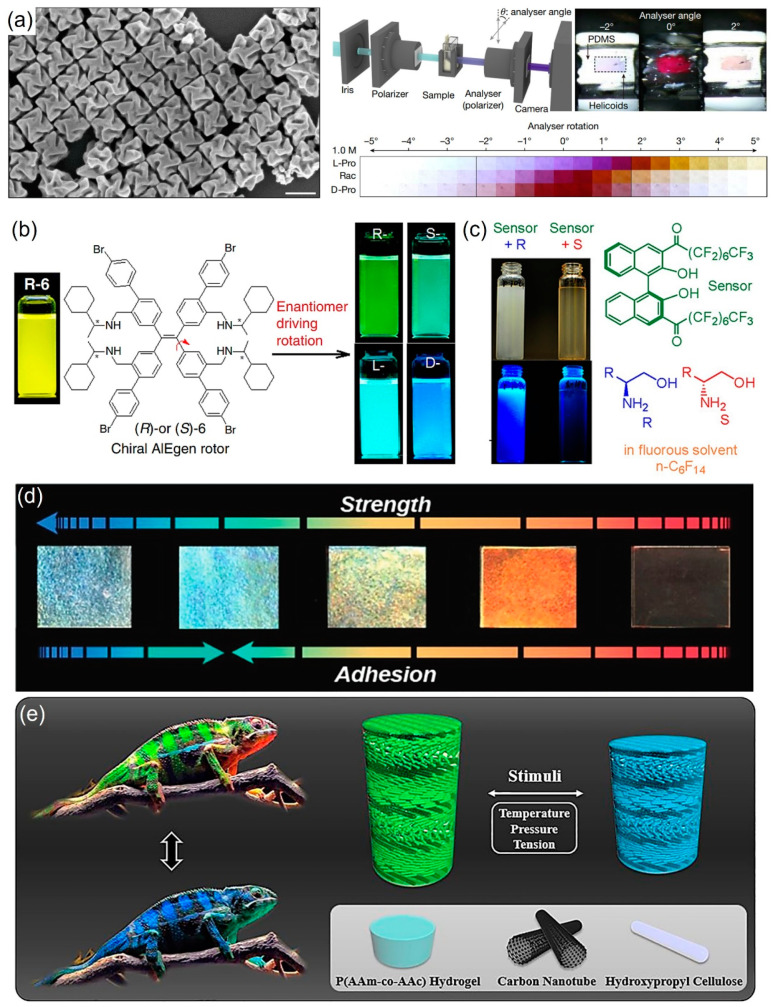
Chiral materials employed for sensing. (**a**) Chiral Au. Reproduced with permission from [60]. Copryright © 2022 Springer Nature Limited. (**b**) chiral aggregation-induced rotor. asterisk in the chemical structure indicates the chiral center. Reproduced with permission from [59]. Copryright © 2020 Springer Nature Limited, and (**c**) BINOL-based enantioselective sensors being able to show clear optical changes depending on the different types of enantiomers. Reproduced with permission from [63]. Copyright © 2015 American Chemical Society. (**d**) Concurrent change of structural color, adhesion strength, and relative humidity of CNC-based composite having Bouligand structure. Reproduced with permission from [10]. Copyright © 2021 John Wiley & Sons. (**e**) Schematic for the electrically conducting CNC-based hydrogel showing pressure and temperature responsive structural color shift pitch distance change of its Bouligand structure. Reproduced with permission from [64]. Copyright © 2020 National Academy of Sciences.

Nam and co-workers presented findings that enantioselective collective resonances can be generated from chiral gold NPs (Figure 6a) [60]. The collective helicoid dipole spinning creates an electromagnetic field having a uniform distribution of the optical helicity density on each two-dimensional crystal plane. The intensity of molecular back action on the electromagnetic field having a uniform distribution of the optical helicity density was different depending on the used enantiomers, providing a spectral difference in circular dichroism. Also, thanks to the optical rotation ability of chiral gold NPs, enantiomers could be visually recognized by chiral gold NPs.

For fluorescent material-based enantioselective sensors, accurate sensing in a highly repeatable manner is hard to achieve due to the liability of fluorescence intensity [59]. Zheng and co-workers demonstrated numerous carboxy group-containing enantiomers can be optically as well as spectroscopically detected by a chiral aggregation-induced emission rotor (Figure 6b) [59]. The emission wavelength of the fluorescent sensor was shifted by the composition of enantiomers, rendering precise detection of the composition. Additionally, an obvious visible color difference was observed between enantiomers in the presence of a chiral aggregation-induced emission rotor, which is ascribed to different wavelength changes caused by different rotations of the rotor depending on the enantiomers. Also, a BINOL (1,1′-bi-2-naphthol)-based chiral diketone can be utilized for enantioselective fluorescent sensors (Figure 6c) [61]. A chiral amino alcohol or diamine-analyte at a concentration higher than 1 mM can increase fluorescent intensity up to 1200–2000 times, whereas its enantiomer only enhances the fluorescent intensity by 10–50 times, enabling the visual discrimination between enantiomers. Spectroscopic investigations revealed that the fluorescent increase and enantioselectivity should have originated from the fluorous solvent-fostered nucleophilic addition of the amino alcohol analytes to the carbonyl groups of the BINOL-based chiral diketone sensor. Dynamic light scattering studies show that there is also a huge difference in particle size when enantiomers aggregate with the BINOL-based chiral diketone sensor.

One of the mesmerizing properties of a chiral nematic structure is that it can show a structural color due to the periodicity of the layers, producing pitch distance and thus Bragg reflection. [61,62]. Kim et al. presented a photonic bio-adhesive that can monitor not only adhesion strength but also humidity changes through visible color changes, using a self-assembled CNC composite that has a chiral nematic structure (Figure 6d) [41]. As the humidity increases, the absorbance of the water molecule at the surface as well as inside of the composite increases, resulting in a reduction of adhesion strength and concurrent increase of pitch distance. As pitch distance increases, the structural color changes in a redshift. This unique correlation enables the real-time colorimetric monitoring of adhesion strength and humidity changes. They further demonstrated that the photonic adhesives can be effectively utilized for not only smart wound dressings that can visually monitor the wound healing process, but also colorimetric sensors that can detect contamination of the surface of respiratory masks. Interestingly, Zhao and co-workers reported that hydroxypropyl cellulose can be self-assembled into a chiral nematic structure in agel composed of hydroxypropyl cellulose, poly(acrylamide-*co*-acrylic acid) hydrogel, and a carbon nanotube (Figure 6e) [10]. As the temperature increases, the intra-molecular interaction between the poly(acrylamide-*co*-acrylic acid) chains is weakened. This is followed by an increase in the amount of hydrogen bonding of the *co*-polymer with water molecules, giving rise to a pitch distance increase and thus a redshifting structural color change. Furthermore, when physical pressure is applied to the composite, the pitch distance decreases, leading to a blueshift in structural color. Finally, thanks to the presence of a carbon nanotube, the composite could also detect the presence of pressure on the composite via electrical signal changes.

## 4. Chiro-Electronics

### 4.1. Device Structure with Operating Mechanisms of Photoconductors, Photodiodes, and Phototransistors

#### 4.1.1. Device Structure

**Figure 7:**
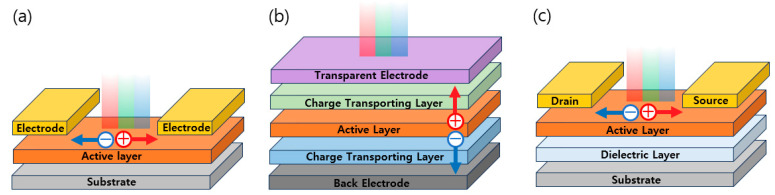
Schematic illustration of representative device structure of CPL detection such as (**a**) Photoconductor, (**b**) Photodiode, and (**c**) Phototransistor.

A Circularly Polarized (CP) photodetector serves as a device that transforms incident circularly polarized light (CPL) into distinct readable electrical signals, contingent upon their circular polarization states. In general, CP photodetectors can be categorized into three types based on their device configuration: photoconductor, photodiode, or phototransistor (Figure 7) [65,66,67]. The sensitivity and CPL discrimination performance of CP photodetectors vary according to the device structure, owing to disparities in the underlying photodetection mechanisms. Consequently, when selecting the appropriate CP photodetector device configuration, one must take into account the surface morphologies, intermolecular interactions, and charge transport characteristics of the CPL-responsive materials.

#### 4.1.2. Operating Mechanisms of Photoconductors, Photodiodes, and Phototransistors

The common configuration of a photoconductor comprises a semiconducting layer that is interfaced with two metallic electrodes featuring ohmic contacts (Figure 7a). In the absence of light illumination, photoconductors manifest elevated resistance owing to the diminished carrier concentration in the semiconducting layers. When subjected to CPL, photo-generated carriers emerge within the semiconducting active layer, which attains heightened conductivity under appropriate matched illumination conditions. During the device’s operation, a singular type of charge carrier undergoes recirculation between the same two electrodes until it recombines with carriers possessing an opposing charge. These devices commonly demonstrate notable optoelectronic outputs originated from the iterative recirculation of multiple carriers.

Photodiodes adopt the most generic type of two-terminal structure, closely resembling that of photoconductors (Figure 7b). However, their operational principles differ significantly. The working mechanism of photodiodes unfolds as follows: Initially, when exposed to CPL, the semiconductor layer produces excitons (electron–hole pairs) with the photoelectric effect. Subsequently, these excitons undergo dissociation into holes and electrons under electric polarization, inducing a current. It is especially important to note that external electrodes gather the free charges with the form of photocurrent. Photodiodes can function in either photovoltaic or photoconductive mode, depending on whether they operate with zero bias or reverse bias, respectively. For the photovoltaic mode operation, where zero bias is applied, one can observe a low-noise and appropriate dark current, while photoconductive-mode operation under reverse bias yields enhanced photoresponsivity (*R*) with a broader depletion region, which indicates the capacity of CPL detection to convert optical input into a readable electrical signal:

$$R=\frac{I_{light}-I_{dark}}{P_{inc}}=\frac{I_{ph}}{P_{inc}}$$

in which $E_{light}$ is the input energy power of the incident light.

In addition, due to unavoidable charge recombination, the external quantum efficiency (*EQE*) of common photodiodes is constrained to 100% and lower values compared to other photodetectors, [68,69]. The ratio between the count of photogenerated carriers amplifying the drain current and photons exposing the channel area is considered:

$$EQE=\frac{I_{ph}hc}{P_{inc}e\lambda }$$

in which h represents the Planck constant, c indicates the speed of light, e denotes the fundamental unit of charge, and λ signifies the maximum absorbance wavelength of the incident light [70]. Consequently, the device’s configuration is an imperative point, taking into consideration the energy band levels and compatible properties with light-harvesting, transporting, and electrode elements.

Phototransistors embody typical three-terminal configurations within field-effect transistors, consisting of the source, drain, and gate electrodes (Figure 7c). In the absence of light, the modulation of channel current between the source and drain is controlled by the applied gate electrode. When subjected to CPL, incident light tunes the channel resistance and charge injection barrier, where it prefers in-plane charge carrier transport of semiconductors, featuring an edge-on molecular orientation. The exposure boosts the carrier concentration of the active layer, amplifying the output photocurrent and *EQE*. Also, depending on the chiral structure of the active materials, they can detect the type of CPL, such as R-CPL and L-CPL, under different polarizations of the incident light. However, during operation, bias stress and interfacial defects can generate charge trapping and residual polarized states. It is advantageous to analyze the transfer graphs with in-situ measurements under illumination to avoid misunderstandings of unintentionally generated charges.

### 4.2. Important Parameters of Circularly Polarized Light in Optoelectronics

Basically, regardless of its polarization state, the chiral semiconducting layer of the detectors exhibits an active condition under light illumination. The photo-generated carriers induce a photocurrent using the absorbed photon energy with the gate bias. For CPL detection, the detector can determine the polarization state of the exposed CPL, then producing different output currents. To achieve a high-performance CPL detector, it is meaningful to consider improving the *g_abs_* (absorption dissymmetry) of the active materials originating from molecular chirality. It can be enhanced by balancing magnetic and electron transition dipole moments [71,72,73]. In terms of hierarchical chirality, it can be generated by inter- and intramolecular interactions, related to the rotational strength ($R_{1,2}^{\prime }$) according to [74]:

$$R_{1,2}^{\prime }\propto \;\pm \;\vec{r_{1,2}}\cdot \vec{\mu_{1}}\times \vec{\mu_{2}}$$

in which $r_{1,2}$, $\mu_{1}$, and $\mu_{2}$ indicate intermolecular distance and the electric transition dipole moment vectors of each molecule, respectively.

The resultant highly-interactive materials, which have a twisted or helical alignment, show amplified chirality following the “sergeant-and-soldiers” and “majority rules”, resulting in a high chiroptical response [75,76]. The chirality in supramolecular systems generates differential absorption under L-CPL and R-CPL, resulting in distinct numbers of photogenerated carriers and the dissymmetry factor ($g_{ph}$) of the performance of CPL detection: [77,78].

$$g_{ph}=2(\frac{I_{L}-I_{R}}{I_{L}+I_{R}})$$

in which $I_{L}$ and $I_{R}$ indicate the photo-generated current upon L-CPL and R-CPL irradiation.

However, some studies reveal an exception that shows s mismatch between $g_{ph}$ of the CPL detection and *g_abs_* of the active layer [79,80]. There is an unintentional dependence of the threshold voltage shift on the handedness, device configuration effect, orbital angular momentum, and spin selectivity [81,82]. Still, the understanding and clarification of concealed factors and individual effects on CPL detection have not been sufficiently considered, which is a question that needs to be investigated from different perspectives.

Simultaneously, the effect of the structure torsion and incorporation of chiral additives to the device performance should not be overlooked, considering the photo-generated charge carrier related to parameters such as responsivity, detectivity, and response time. The *pn* junction must occur below a specific threshold to block interference with chirality of the composites. To mitigate this adverse impact, a small portion of the *p*- or *n*-counterparts might generate the photomultiplication effect and charge trapping [83]. Therefore, the several strategies of designing device structures should be studied by introducing liquid crystalline materials, optimized heterostructures, and doping treatment to improve the charge carrier extraction and $g_{ph}$.

### 4.3. Strategies of CPL Detection for High Performance

Beyond the perspective of device configuration, it is important to consider other strategies such as materials, composites, and doping for improving high CPL detection. Chiral materials exhibit inherent asymmetry, which makes it hard to overlay their mirror-image counterparts. Specifically, chiral semiconductors can detect CPL directly through interactions between light and active materials. They can eliminate the need for additional optical components, serving as viable active elements within both chiroptical and chiral optoelectronic devices.

Despite notable advancements in CPL photodetectors over recent years, there remain critical issues to be resolved for practical implementation, such as the performance of CPL detectors. Consequently, various strategies have been employed to enhance the performance of CPL photodetectors, encompassing cholesteric liquid crystals (CLC), heterostructures, and doping techniques.

#### 4.3.1. Cholesteric Liquid Crystals

CLCs show organized LC formations characterized by intrinsic periodicity and adopting helical supramolecular structures. They are applicable to tunable color filters, diffraction gratings, and reflective optics [84,85]. The interesting cholesteric nematic structures recommend an effective strategy to enhance CPL photodetector performance. Specifically, they have exhibited robust selective chiro-optical properties, modulating the reflection and the transmission of CPL with the same and opposite chirality, respectively [86].

**Figure 8:**
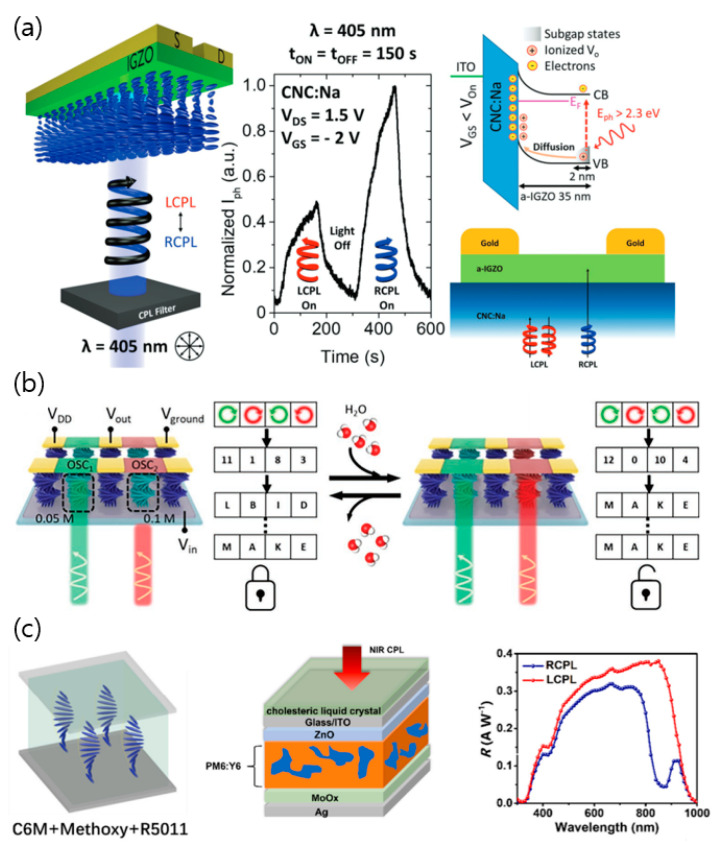
Representative CPL detector by using CLC. (**a**) CPL detection of a-IGZO transistor by facilitating selective transmission of chiral nematic structure of CNC films. Reproduced with permission from [89]. Copyright © 2020 AIP Publishing LLC. (**b**) Encryption application of CNC-based integrated inverter circuit responsive to CPL, humidity, salt concentration. Reproduced with permission from [90]. Copyright © 2019 John Wiley & Sons. (**c**) NIR CPL sensing of organic phototransistors by CLCN films. Reproduced with permission from [92]. Copyright © 2018 John Wiley & Sons.

Moreover, stacking CLC network films into optoelectronics based on a cross-linking process is a compatible processing option. For example, cellulose nanocrystals (CNC) exhibit biocompatibility, renewability, and cost-effectiveness, possessing the left-handed chiral nematic configuration even in the film state, and they offer the evaporation-induced self-assembly [87,88]. They have shown capabilities when integrated into CPL-detectable devices. For example, Pereira et al. fabricated amorphous indium-gallium-zinc-oxide (a-IGZO)-based field-effect transistors (FET) integrated with a CNC gate dielectric layer (Figure 8a) [89]. They demonstrated that approximately 40% of L-CPL was reflected by the CNC composite layer with a transmission of ~95% to the semiconducting element, inducing selective post-photocurrent depending on CPL. It allowed the sensors to differentiate between the two circular polarization states and converted CPL to discrete electrical signals. Moreover, Han et al. developed a CNC composite film as an electrolyte layer within the FET system. The film was responsive to humidity and CPL (Figure 8b) [8,90,91]. Basically, they could modulate the transmission properties depending on the humidity, where chiral pitch increased as water molecules were intercalated with a humidity increase. By facilitating the tunable optical properties, the subsequent electrical properties showed multivalued-logic signals beyond the generic binary systems. Also, by selective ink-jet printing of a salt solution onto the prepared CNC composite films, tunable optoelectronic signals can be generated under light irradiation of different chiral polarization states, then passed to the integrated inverter circuits. Therefore, they can convert photon energy and photonic energy as well as CPL information into readable electronic signals, applicable to encryption devices.

Employing cholesteric liquid crystal network films (CLCN), researchers investigated a near-infrared circularly polarized light–sensing photodetector (NIR CPL-OPTR) (Figure 8c) [92]. Based on the chiral dopant types, the CLCN film’s helical supramolecular structure tuned the polarization orientation of CPL, resulting in the NIR CPL-OPTR device achieving a dissymmetry factor of 1.56. Additionally, both of the maximum *R* reached 0.36 A W^−1^ at 0.1 V, 7.12 × 10^12^ of detectivity, 127.6 dB of linear response (LDR), and rise and decay times of 0.65 and 5.7 ms, respectively. Moreover, the NIR CPL-OPTR arrays were applied to be a physically unclonable function device to improve chiro-optoelectronic cryptographic primitives [93].

#### 4.3.2. Heterojunction via Chiral Templating and Doping Treatment

**Figure 9:**
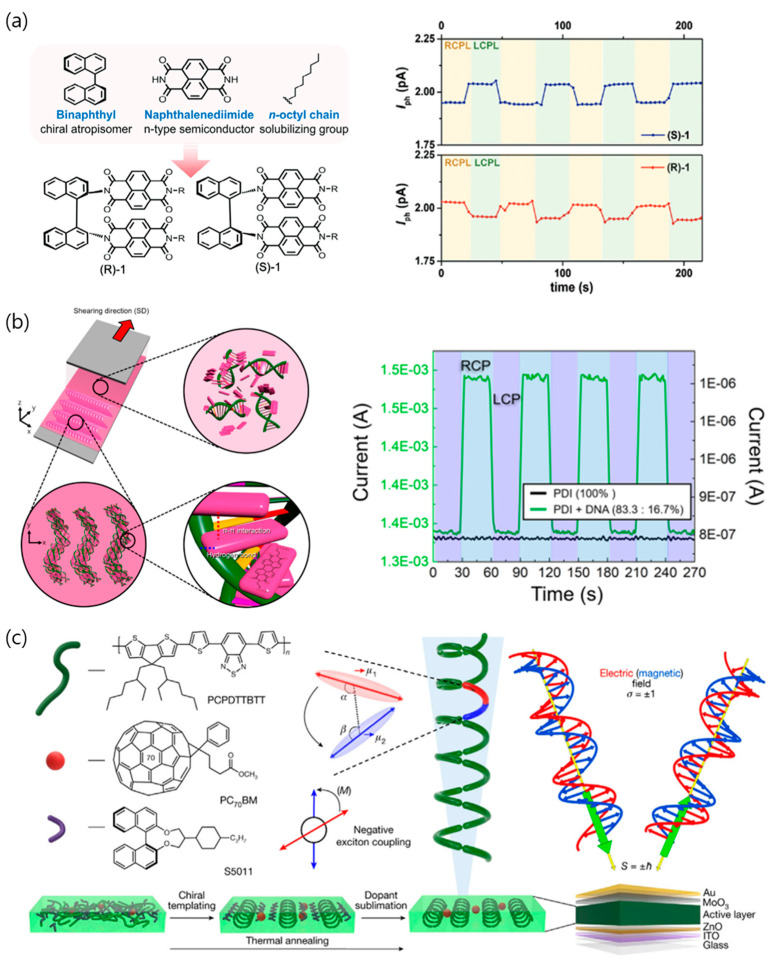
Representative illustration of heterojunction to induce chirality for CPL detection. (**a**) NDI/BINAM (R or S) molecular design to induce chiral self-assembly behavior, operating CPL detection of phototransistor. Reproduced with permission from [96]. Copyright © 2019 John Wiley & Sons. (**b**) Schematic illustration of the induced chiral structure of DNA-PDI complex by evaporation-induced self-assembly process, responsive to the incident CPL. Reproduced with permission from [97]. Copyright © 2022 American Chemical Society. (**c**) Chiral dopant-assisted chiral structure of semiconducting polymer to fabricate CP photodetector. Reproduced with permission from [98]. Copyright © 2023 John Wiley & Sons.

Fundamental advancements in material systems and device structures are crucial to surmount inherent limitations in CPL detection. Among them, heterojunctions are a potentially effective approach to improving CPL photodetector performance, which are related to the spatial separation of electron–hole pair separation [94,95]. Kwon et al. introduced naphthalene diimides (NDIs)/BINAM heterojunction active layers to phototransistors, where BINAM acted as a chiral scaffold to induce chiral self-assembly behavior, yielding nanowires (Figure 9a) [96]. Specifically, the gradual decrease of the enantiomeric ratio generated the formation of NPs. The organic photodetector based on thermal deposition showed a relatively high electron mobility of 0.22 cm^2^ V^−1^ s^−1^ and 0.05 of *g_ph_*. Likewise, Han et al. manipulated DNA-templated achiral PDI aggregates using a solution shearing technique (Figure 9b) [97]. Semiconducting PDI molecules were engaged with DNA via hydrogen-bond and π-π interactions, showing a hierarchically chiral organization under the influence of the elastic energy of DNA. The optimized ratio of DNA to the composite films and shearing speed generated a noteworthy electron mobility of up to 3.97 cm^2^ V^−1^ s^−1^ coupled with a substantial $g_{ph}$ of + 0.14 at 465 nm.

In recent developments, highly chiroptical films were demonstrated by incorporating chiral dopants of 1,10-bi-2-naphthol derivatives (R5011 and S5011) into a semiconductor of poly[2,6-(4,4-bis(2-ethylhexyl)-4H-cyclo-penta-[2,1-b:3,4-b0]dithiophene)-alt-4,7-bis(thiophene-2-yl) benzo-2,1,3-thiadiazole]. (PCPDTTBTT) (Figure 9c) [98]. Thermal annealing with dopant sublimation generated the chiral orientation of the guest semiconducting polymer. Ultimately, a dissymmetry factor of 1.2 was reached, representing the highest dissymmetry factor obtained in an organic photodiode.

Numerous studies have investigated the potential of molecular doping treatments, especially surface doping, to optimize opto-electronic properties by introducing electron donors or acceptors to adjust Fermi levels [99,100]. Shang et al. fabricated single crystals of (R)-C1CPDI-Ph-CF and (R)-C1CPDI-DMF, which were applied to a CPL phototransistor [101]. The additional surface doping with hydrazine improved the optoelectronic properties compared to the pristine single crystal case, which aligned with the Density functional theory (DFT) results derived from the increased electron affinity following hydrazine absorption.

## 5. Concluding Remarks and Perspective

Obviously, chiral materials have paved the way for advancing optics and electronics. But at the same time, clearly the research into optics and electronics enabled by chiral materials have not matured considering following two big remaining tasks.

First, employing more diverse chiral materials to optics and electronics: While chiral materials have been rapidly put to use in optics and electronics, limited types of chiral materials have been used, primarily amino acids, peptides, DNA, BINOL, and LC. Therefore, by applying more varieties of chiral materials, the performance of chiral materials to optics and electronics could be further investigated and thus could further advance the development of optics and electronics.

Second, development of various chiral materials and application of them to optics and electronics: To apply diverse chiral materials to optics and electronics, it is vital to further develop the kinds of chiral materials. The control of a material’s chirality has been a significantly important research topic, and it has challenged researchers, who have accordingly been rewarded. For instance, the Nobel Prize in Chemistry 2021 was awarded to David Macmillan and Benjamin List for the development of asymmetric organocatalysis [3]. Also, the Nobel Prize in Chemistry 2001 was awarded to K. Barry Sharpless for his work on chirally catalysed oxidation reactions and to William S. Knowles and Ryoji Noyori for their work on chirally catalyzed hydrogenation reactions [102]. Among diverse research projects on the control of a material’s chirality, the synthesis of chiral materials from achiral precursors is of interest, considering the abundance of achiral materials that would thus have a high potential utility in generating numerous chiral materials [30].

Compared to the general photodetector, CP photodetectors based on chiral materials are attracting significant interest as promising platforms in optoelectronics for diverse applications, including high-resolution imaging and biosensing. This growing attention stems from their ability to enhance the transfer of information during interactions between light and matter, thereby improving memory retrieval. Additionally, CPL emitters offer new perspectives for 3D displays by presenting distinct images to each eye using LCPL and RCPL. These unique characteristics are advantageous in augmented/virtual reality systems and displays with high brightness, reducing the losses seen in traditional optical filters. Intrinsic CPL generation enables the differential transmission of light through polarization lenses, essential for the anti-glare filters and binocular disparity of 3D visualizations, while preserving display brightness. Furthermore, the combined use of CP photodetectors and emitters allows for polarization multiplexing in optical communication channels, exploiting polarization-based encoding for secure data transmission. This method could prove to be valuable in multi-channel data processing, CPL-encoded optical communications, and the secure transfer of highly encrypted information.

Overall, through continuous effort to develop methods of synthesizing various chiral materials and concurrently using them in optics and electronics, we expect that we can further advance optics and electronics, which will lead us to places that we have never been before and expand the frontier of human knowledge.

## Data Availability

Not applicable.
